# MicroRNA-29a increased the intestinal membrane permeability of colonic epithelial cells in irritable bowel syndrome rats

**DOI:** 10.18632/oncotarget.20687

**Published:** 2017-09-06

**Authors:** Guanqun Chao, Yingying Wang, Shuo Zhang, Weilin Yang, Zheying Ni, Xuliang Zheng

**Affiliations:** ^1^ Department of Gastroenterology, The First Affiliated Hospital, Zhejiang Chinese Medical University, Zhejiang, China; ^2^ Department of Family Medicine, Sir Run Run Shaw Hospital, Zhejiang University, Zhejiang, China

**Keywords:** diarrhea-predominant irritable bowel syndrome (IBS-D), microRNA-29a, intestinal membrane permeability, aquaporins (AQPs)

## Abstract

**Background:**

The whole pathogenesis of diarrhea-predominant irritable bowel syndrome(IBS-D) is poorly understood. Our goal was to evaluate the expression change of microRNA-29a(miR-29a) in colonic epithelial cells in IBS rats and clarify the mechanism of miR-29a increasing the intestinal membrane permeability through aquaporins(AQPs).

**Methods:**

The IBS-D rats models were induced by rectal distention pressure combining with extremities constraint. The colonic epithelial cells were divided into four groups. A: normal group. B: IBS-D control group. C: IBS-D +miR-29a NC. D: IBS-D + miR-29a antagomir. The expression of miR-29a, the concentration of the K^+^ and Lactate Dehydrogenase(LDH) and the expression of AQPs were detected.

**Results:**

The miR-29a expression increased in IBS-D control group(2.090±0.022) compared with the control group(1.00±0.031) (P<0.001) while it decreased in IBS-D+miR-29a antagomir group(1.403±0.042) compared with IBS-D control group(P<0.001). The K^+^ decreased in IBS-D control group(1.305±0.289) compared with the control group(2.171±0.204)(P<0.05) while it increased in IBS-D+miR-29a antagomir group(1.813±0.102)(P<0.05) compared with IBS-D control group. The LDH increased in IBS-D control group(4153.440±177.365) compared with the control group(1434.573±96.111)(P<0.001) while it decreased in IBS-D+miR-29a antagomir group(2700.473±275.414) compared with IBS-D control group (P<0.01). The expression of AQP1, AQP3 and AQP8 decreased in IBS-D control group(0.132±0.010,0.110±0.005,0.108±0.007) compared with the control group (P<0.001) while it increased in IBS-D+miR-29a antagomir group(0.197±0.005,0.182±0.011,0.194±0.003) compared with IBS-D control group(P<0.001). The IBS-D+miR-29a negative control(NC) group, a comparison with IBS-D+miR-29a antagomir group, each date showed the similar trend to the IBS-D control group.

**Conclusions:**

MiR-29a increased the intestinal membrane permeability of colonic epithelial cells by reducing the AQPs expression in IBS-D rats.

## INTRODUCTION

Irritable bowel syndrome (IBS) is the most common functional gastrointestinal diseases which affects up to 10%-20% [1]. The main clinical symptoms of IBS are abdominal pain, distension or abdominal discomfort and the change of defecation habits. The whole pathophysiological mechanisms underlying symptoms in IBS stay unknown [2, 3]. It is regarded as a multifactorial condition that affects individuals differentially [4].Actually, gastrointestinal diseases are often concerned with the increase of intestinal permeability when diarrhea and abdominal pain occur [5]. Growing evidence has shown that patients with diarrhea-predominant IBS have increased intestinal membrane permeability [6–9], and studies showed the permeability of small intestine and colon both increase [9, 10]. The complete mechanism leading to the increase of intestinal membrane permeability still needs further study.

Aquaporins (AQPs) have the function of transporting water or other small solutes [11–12], which play an important role in water balance. Fecal characteristics are closely related with intestinal fluid metabolism, the pulpy feces in diarrhea-predominant IBS are the performance of disorders of water metabolism. Recently, studies confirmed AQP1, AQP3 and AQP8 are closely related with water transportation in colon [13, 14]. Research considered the decreased expression of AQP8 in D-IBS indicated the damage of colonic absorption function, which led to the diarrhea [15]. In addition, some studies confirmed that AQPs take part in the composition of the intestinal mucosal barrier, and the decrease of AQPs resulted in the increase of intestinal membrane permeability. Scholars found that knockdown of aquaporin3 was involved in intestinal barrier integrity impairment, which influenced the intestinal membrane permeability [16]. Thus, we speculate that the lack of AQPs which affects the intestinal membrane permeability may be the key of the occurrence of symptoms.

MicroRNAs (miRNAs) are a class of 18–24 nucleotide long non-coding RNA molecules with control function. MiRNAs combine with the 3'-UTR of the target mRNA, causing degradation of target genes or inhibiting the activity of translation through complementary identification, to achieve regulation of target gene expression [17]. Till now, thousands of miRNAs have been found, which make a difference in the process of metabolism, proliferation, apoptosis and differentiation of cells. Studies showed that miRNAs take part in a lot of pathophysiologic processes including inflammation, and its regulation of immune response plays an important role in the process of colony-stimulating factor and pathogen invasion [18]. In recent years, studies on correlation between miRNA and IBS have been concerned, which have shown that alterations of microRNAs (miRNAs) levels have affected IBS, and certificated the role of microRNAs in IBS with increased gut permeability [19–23].

MiRNA-29a is a member of miRNA-29 family. Study displayed the up-regulated miRNA-29 resulted in the increased intestinal permeability [24]. Moreover, miR-29a increased in IBS patients, and researchers conjectured miRNA-29a may effect on intestinal membrane permeability through its regulation of GLUL in IBS [25]. Nonetheless, what proteins regulated by miR-29a to affect the intestinal membrane permeability remains unknown. Since IBS-D is the most part in IBS, the goal of our study was to evaluate the expression of miRNA-29a in colonic epithelial cells in IBS-D rats and clarify the mechanism of miRNA-29a regulating the intestinal membrane permeability by AQP1, AQP3, AQP8.

## RESULTS

### The colonic epithelial cells identification

**Figure 1:**
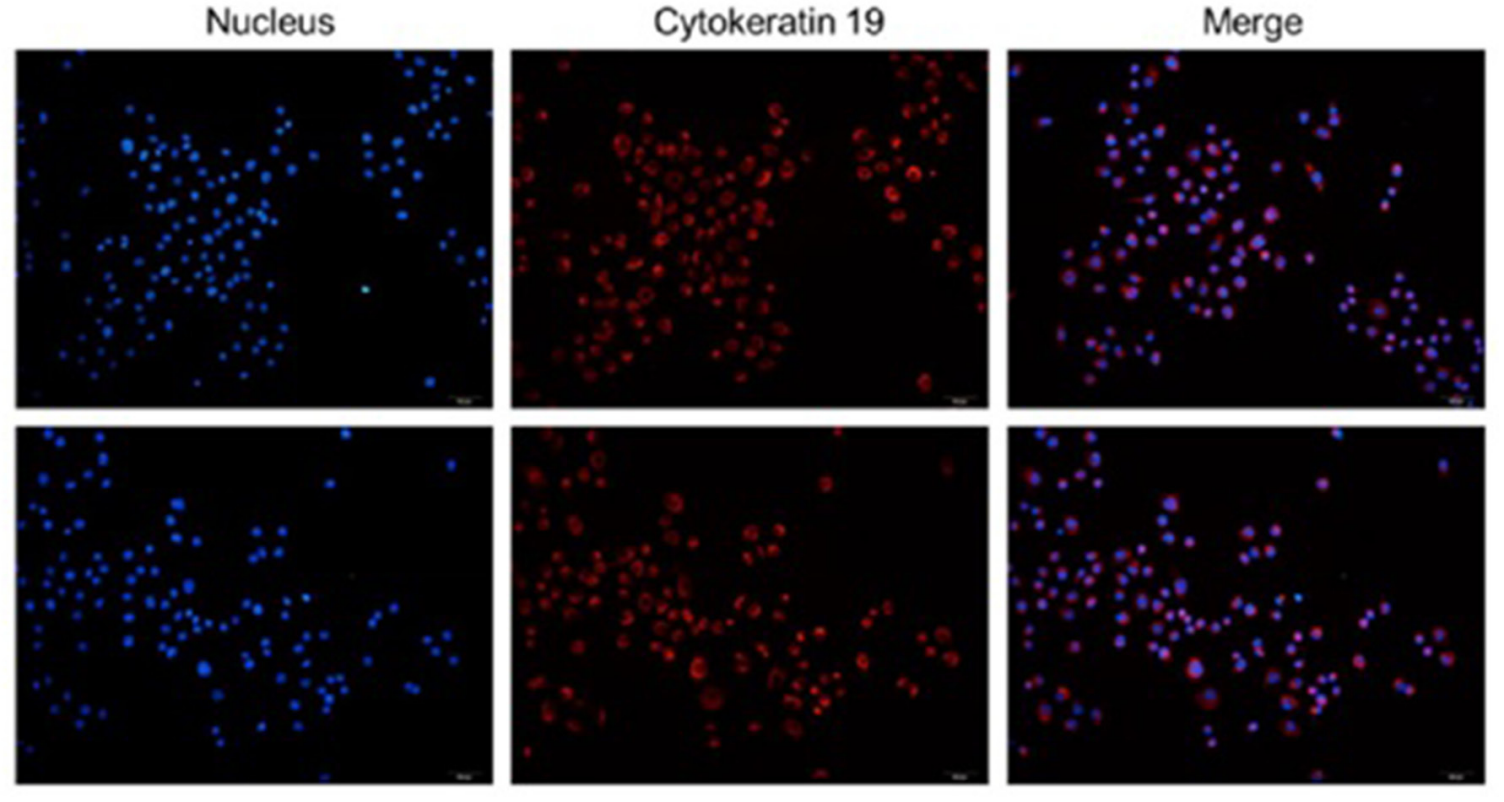
The colonic epithelial cells identification The positive cells rate of Keratin 19≥95%, the colonic epithelial cells could be determine.

The positive cells rate of Keratin 19 ≥ 95%, the colonic epithelial cells could be determined (Figure 1).

### qRT-PCR

**Figure 2:**
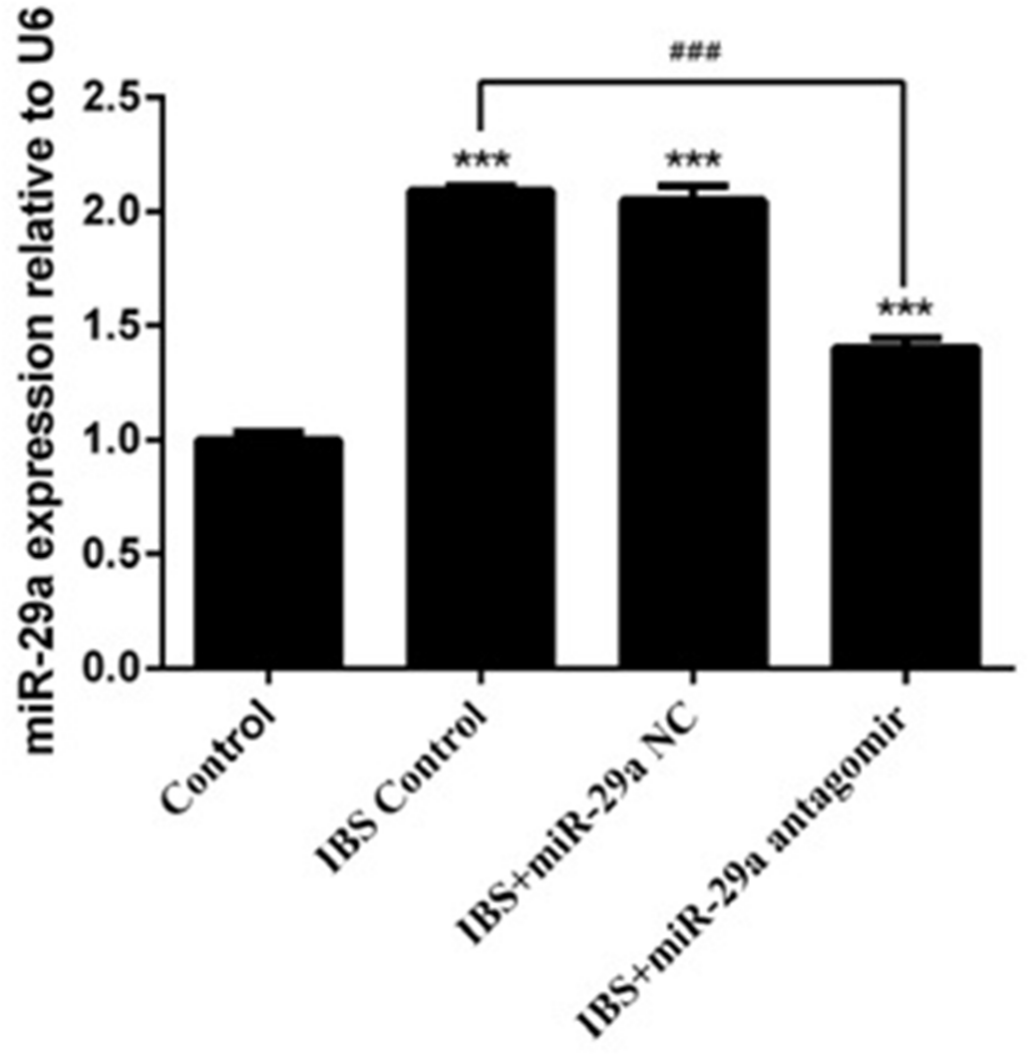
The change of miR-29a expression in the colonic epithelial cells of IBS-D rats and the effect of miRNA-29a antagomir ***P<0.001 vs. Control. ### P<0.001 vs. IBS-D Control. Compared with the control group, the expression of miR-29a increased significantly in IBS-D control group, IBS-D +miR-29a negative control(NC) group, which reflected that miR-29a increased in IBS-D rats.Besides, the miR-29a NC was confirmed to be a great negative control in related to miR-29a antagomir. Compared with IBS-D control group, the expression of miR-29a decreased while miR-29a antagomir added, showing an actual function of obliteration.

Compared with the control group(1±0.031), the expression of miR-29a increased significantly in IBS-D control group(2.09±0.022), IBS-D +miR-29a NC group(2.047±0.064) and IBS-D+miR-29a antagomir group(1.403±0.042), showing extremely differences(P<0.001), which showed the expression of miR-29a increased in IBS-D rats. Compared with IBS-D control group(2.09±0.022), the expression of miR-29a decreased significantly in IBS-D+miR-29a antagomir group(1.403±0.042), showing extremely differences (P<0.001), which indicated antagomir blocked out the expression of miR-29a (Figure 2).

### The concentration of K^+^

**Figure 3:**
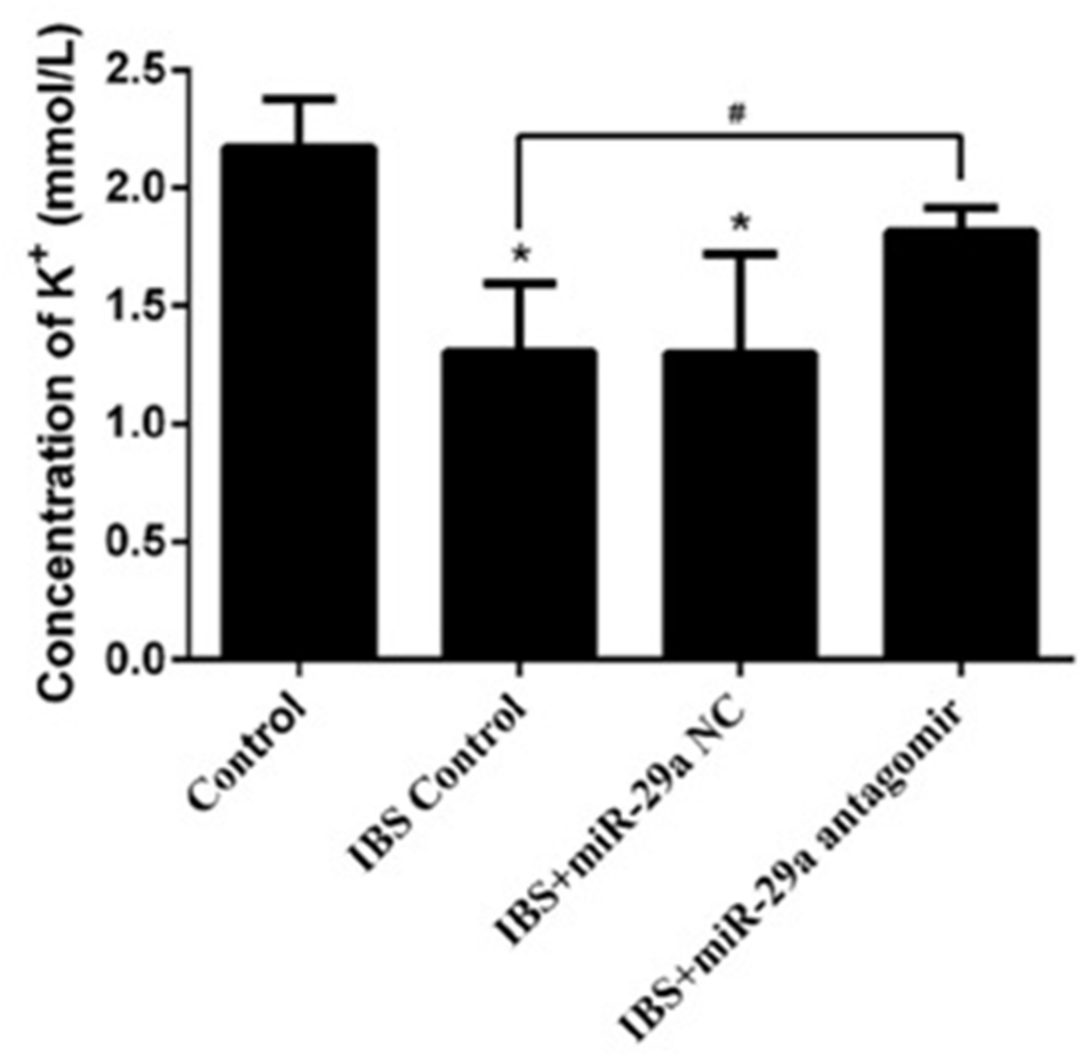
MiRNA-29a regulated concentration of K^+^ in the colonic epithelial cells of IBS-D *P<0.05 vs. Control. #P<0.05 vs. IBS-D Control. Compared with the control group, the concentration of K^+^ decreased significantly in IBS-D control group, IBS-D +miR-29a NC groupCompared with IBS-D control group, the concentration of K^+^ increased significantly in IBS-D +miR-29a antagomir group. The concentration of K^+^ change proved the intestinal membrane permeability inceased significantly with the miR-29a increasing in the IBS-D rats, showing the real function of miR-29a in regulating the intestinal membrane permeability.

Compared with the control group(2.171±0.204), the concentration of K^+^ decreased significantly in IBS-D control group(1.305±0.289), IBS-D +miR-29a NC group(1.299±0.420) and IBS-D+ miR-29a antagomir group(1.813±0.102), and there were significant differences in IBS-D control group and IBS-D+miR-29a NC group (P<0.05) while there were no significant differences in IBS-D+miR-29a antagomir group(P>0.05). Compared with IBS-D control group, the concentration of K^+^ increased significantly in IBS-D +miR-29a antagomir group (P<0.05) (Figure 3). The above date showed the higher expression of miR-29a lead to the decreased of concentration of K^+^ which is a sign of intestinal membrane permeability.

### The concentration of LDH

**Figure 4:**
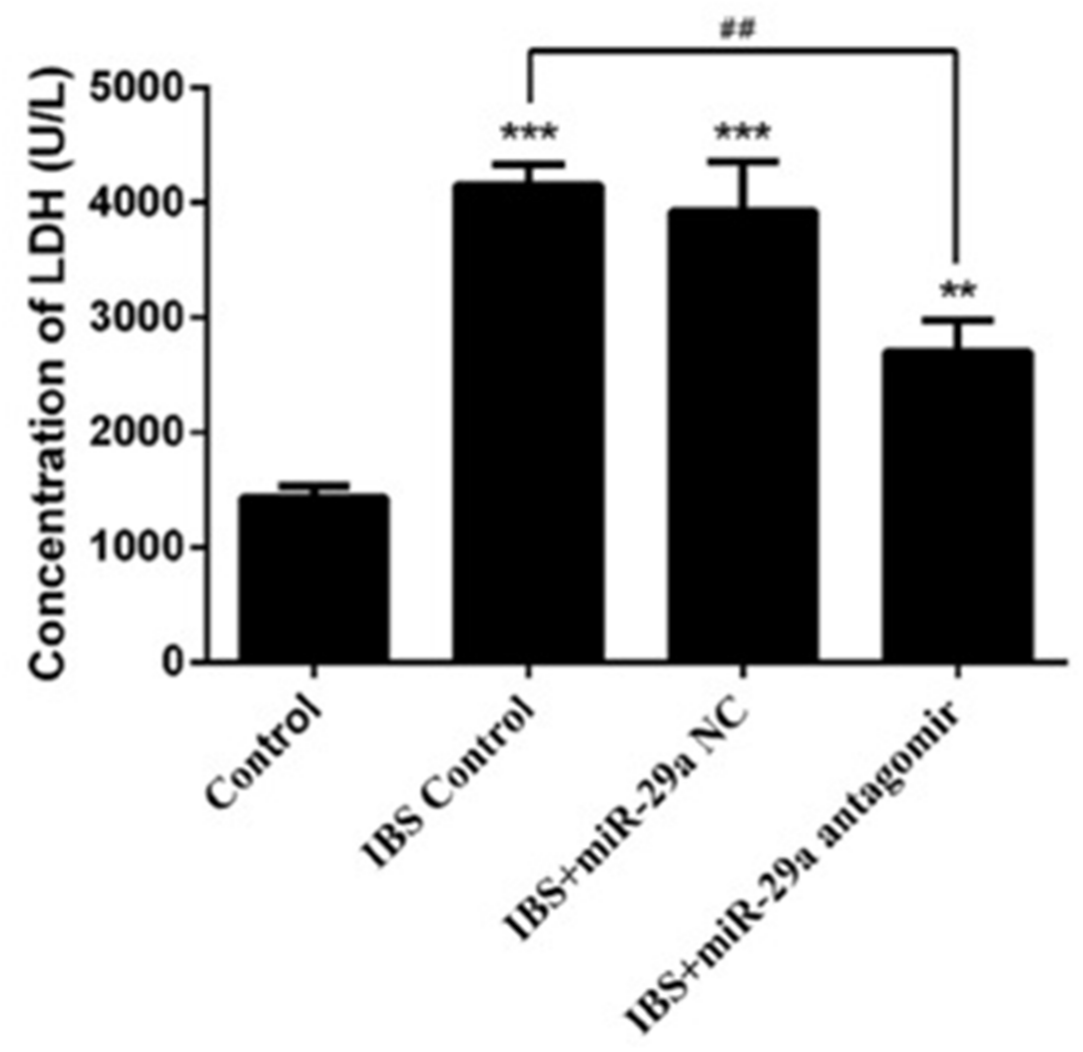
MiRNA-29a regulated concentration of LDH in the colonic epithelial cells of IBS-D **P<0.01, ***P<0.001 vs. Control. ##P<0.01 vs. IBS-D Control. Compared with the control group, the concentration of LDH increased significantly in IBS-D control group, IBS-D +miR-29a NC group Compared with IBS-D control group, the concentration of LDH decreased significantly in IBS-D+miR-29a antagomir group The concentration of LDH change was another evidence to prove the intestinal membrane permeability inceased significantly in IBS-D rats. Also, the function of miR-29a in regulating the intestinal membrane permeability was confirmed.

Compared with the control group(1434.573±96.111), the concentration of LDH increased significantly in IBS-D control group(4153.440±177.365), IBS-D +miR-29a NC group(3924.384±438.095) and IBS-D+miR-29a antagomir group(2700.473±275.414), showing extremely differences(IBS-D control group, IBS-D+miR-29a NC group P<0.001, IBS-D+miR-29a antagomir group P<0.01). Compared with IBS-D control group(4153.440±177.365), the concentration of LDH decreased significantly in IBS-D+miR-29a antagomir group(2700.473±275.414), showing extremely differences(P<0.01)(Figure 4). As a mark of intestinal membrane permeability, the increased concentration of LDH denoted the increased intestinal membrane permeability. Hence, miR-29a influenced the permeability, as the higher expression of miR-29a in the IBS-D control group and IBS-D+miR-29a NC group lead to the higher leakage rates of LDH, showing positive correlation.

### Western blot

Compared with the control group(0.229±0.013), the expression of AQP1 decreased significantly in IBS-D control group(0.132±0.010), IBS-D +miR-29a NC group(0.124±0.010) and IBS-D+ miR-29a antagomir group(0.197±0.005), showing extremely differences(IBS-D control group, IBS-D+miR-29a NC group (P<0.001), IBS-D+ miR-29a antagomir group P<0.05). Compared with IBS-D control group, the expression of AQP1 increased significantly in IBS-D+miR-29a antagomir group (P<0.001).

Compared with the control group(0.221±0.004), the expression of AQP3 decreased significantly in IBS-D control group(0.111±0.005), IBS-D +miR-29a NC group(0.102±0.008) and IBS-D+ miR-29a antagomir group(0.182±0.011), showing extremely differences(IBS-D control group and IBS-D+miR-29a NC group P<0.001, IBS-D+ miR-29a antagomir group P<0.01). Compared with IBS-D control group, the expression of AQP3 increased significantly in IBS-D+miR-29a antagomir group(P<0.001).

**Figure 5:**
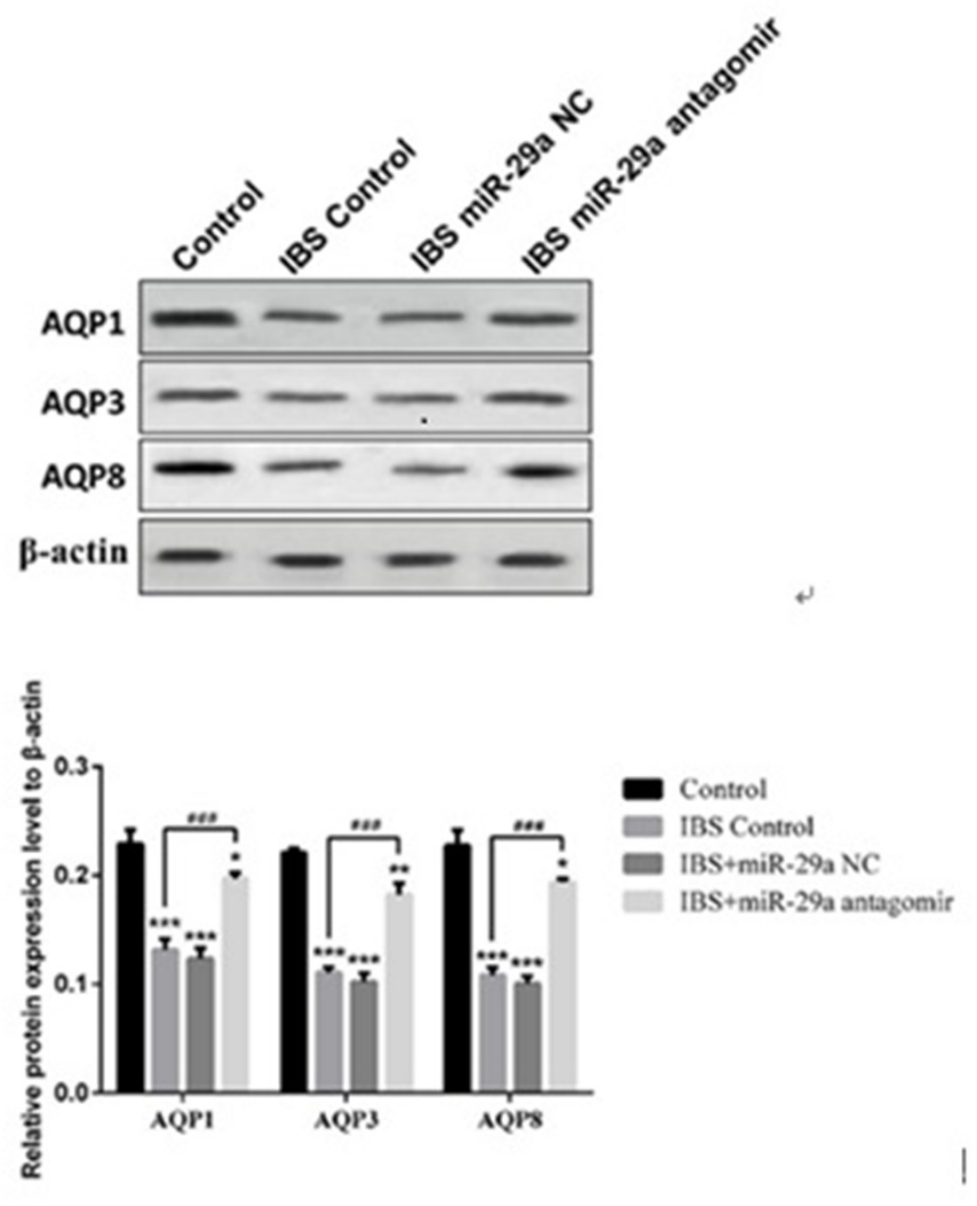
MiRNA-29a regulated AQP1, AQP3, AQP8 in the colonic epithelial cells of IBS-D *P<0.05, **P<0.01, ***P<0.001 vs. Control. ###P<0.001 vs. IBS-D Control. Compared with the control group, the expression of AQPs decreased significantly in IBS-D control group, IBS-D +miR-29a NC group. Compared with IBS-D control group, the expression of AQPs increased significantly in IBS-D+miR-29a antagomir group. The results showed the expression of AQP1, AQP3 and AQP8 all decreased together with the increase of intestinal permeability in IBS-D rat colon which reflected that AQPs may be the key of the change of intestinal permeability.

Compared with the control group(0.227±0.014), the expression of AQP8 decreased significantly in IBS-D control group(0.108±0.007), IBS-D+miR-29a NC group(0.101±0.007) and IBS-D+ miR-29a antagomir group(0.194±0.003), showing extremely differences(IBS-D control group and IBS-D+miR-29a NC group P<0.001, IBS-D+ miR-29a antagomir group P<0.05). Compared with IBS-D control group(, the expression of AQP8 increased significantly in IBS-D+miR-29a antagomir group(P<0.001) (Figure 5).

In IBS-D rats, AQPs’ expression all decreased. On the contrary, while miR-29a antagomir added, the expression increased compared with IBS-D control group, indicating miR-29a regulated the expression of AQPs, which showing negative correlation.

## DISCUSSION

Growing evidence has shown that patients with diarrhea-predominant IBS(IBS-D) have increased intestinal membrane permeability [6–9]. MiR-29a has been confirmed to take part in the regulation of intestinal membrane permeability in IBS. However, how miR-29a regulates the permeability is only partly known yet. Our study proved that diarrhea-predominant IBS rats have increased intestinal permeability which is associated with the increase of miR-29a expression in colon epithelial cells. Besides, the study also provided evidence to confirm the up-regulation of miR-29a expression in colon may increase the intestinal membrane permeability in IBS rats through the down-regulation of AQPs(AQP1, AQP3, AQP8).

Mucosal barrier, constituted by intestinal epithelial cells and the junctions, played an important role in keeping homeostasis and health. Studies showed alterations of mucosal barrier function were related to a great variety of disorders which were linked to the intestine, including inflammatory bowel disease (IBD), irritable bowel syndrome, metabolic syndrome, etc [26]. Intestinal epithelial cells are held together by adherens junctions, tight junctions (TJs) and desmosomes. In fact, TJs are the keys to regulating the intestinal permeability by allowing the passage of water, ions, and solutes through pores [27]. As a result, destruction of physical barrier is certain to increase the intestinal permeability. Numerous studies confirmed that patients with diarrhea-predominant IBS have increased intestinal membrane permeability [6–9, 28]. The increased mucosal permeability resulting in the visceral hypersensitivity leads to increased gastrointestinal symptoms in IBS [29]. In this study, we tested the concentration of K^+^ and LDH of intestinal epithelial cells, and found that compared with the control group, the concentration of K^+^ decreased while LDH increased obviously in IBS-D rats, which proved intestinal permeability increased significantly in IBS-D rats once again. What’s more, the obliteration of miR-29a led to decreased intestinal permeability contrast to the IBS-D control group, manifesting that miR-29a is a critical factor to regulate intestinal permeability.

The correlation between miRNAs and IBS has been concerned recently. The alternation of miRNAs were found in IBS, and study confirmed the role of miRNAs in IBS with increased gut permeability [19–23]. MiR-29a has been confirmed to take part in the regulation of intestinal membrane permeability in IBS. Zhou [24] found that miR-29 targets on nuclear factor-κB-repressing factor and Claudin 1 to increase intestinal permeability. Moreover, another research shown that altered miR-29a expression in intestine may regulate intestinal permeability in IBS patients through glutamine dependent mechanisms as proved by the functional interaction between miR-29a and the GLUL [25]. All in all, the former studies indicated miR-29a has significant effect on intestinal membrane permeability. Our study found that the higher expression of miR-29a resulted in the increasing of intestinal membrane permeability in IBS-D. Interestingly, compared with IBS-D control group, concentration of K^+^ decreased significantly while concentration of LDH increased significantly in IBS-D+miR-29a antagomir group. All above reflected that miRNA-29a was a key point to regulation of the intestinal membrane permeability. Combined former and this studies, we infer that miRNA-29a influences the intestinal membrane permeability through multiple target.

Zhou considered the increased miR-29a which caused the decreased CLDN1 signaling, which may lead to increased intestinal permeability through several potential mechanistic pathways [24]. In order to explain how miR-29a influences intestinal membrane permeability through potential mechanistic pathways, we focus on AQPs. AQPs exist widely in the intestinal tract, which have an effect on the process of water transportation [30]. The alternations of AQPs on the colon epithelial cells will result in the change of intestinal absorption function, which has a significant influence on waste nature. Studies have verified the change of AQPs in intestine is significant to the diarrhea in IBS [31–32]. AQP1, AQP3 and AQP8 are closely related with water transportation in colon [13, 14]. The former studies focused on the water transportation of AQPs which caused the change of waste nature, but the relationship between AQPs and other symptoms had been ignored. In this study, we detected the expression of AQP1, AQP3, AQP8 in different groups, and got a result that the expression of all of these AQPs decreased in IBS-D rat colon epithelial cells. Interestingly, compared with IBS-D control group, the expression of the three kinds of AQPs was higher in IBS-D+miR-29a antagomir group, which indicated the deceased of miRNA-29a have an up-regulation on AQPs. Thus, we confirmed that the increased expression of miRNA-29a in IBS-D led to the deceased of AQPs, which affected the intestinal permeability. Furthermore, recent studies manifested that AQPs is a part of mucosal barrier, the down-regulation of AQPs in colon will lead to the increase of intestinal permeability. Zhang [33] found that knockdown of AQP3 was associated with a reduction of Claudin-1 and Occludin expression and increase of bacteria translocation, which inferred AQP3 was related with intestinal permeability by the down-regulation of TJs. Our data showed the expression of AQP1, AQP3 and AQP8 all decreased together with the increase of intestinal permeability in IBS-D rat colon. Therefore, we supposed that, similar to AQP3, AQP1 and AQP8 can also regulate the TJs which lead to the change of intestinal permeability in IBS-D and then cause the recurrent abdominal discomfort or pain not only the change of waste nature.

In summary, our study is unique that we concentrated on the protein level in IBS-D on the basis of increased expression of miR-29a. We firstly found the relation between miR-29a and AQPs in the IBS-D colon tissues. Study indicated miR-29a regulated the intestinal permeability target on AQPs. However, we have not detected the expression of TJs, the specific relationship between AQPs and intestinal membrane permeability are needed to be explored in depth. Thus, further studies are needed to demonstrate miR-29a as a treatment target in the IBS-D, and the relationship between AQP1, AQP3, AQP8 and intestinal permeability are supposed to be clarified. Moreover, other targets of miR-29a should also be found in IBS-D to complete the mechanism in the future. However, although miRNAs technique is an exciting treatment for IBS-D, there are also many challenges, including potential missing effects and delivery problems [16]. Finding out biological function of miRNAs, proved its expression and regulation mechanism eventually, will have profound significance for better understanding of IBS-D. In a word, our findings may lead to new therapeutic targets [34] for the treatment of IBS-D with increased intestinal permeability via miR-29 regulation.

## MATERIALS AND METHODS

### Equipment and reagents

Trizol were from invitrogen; SYBRGreen PCR Kit were from Thermo F-415XL; reverse transcription Kit were from Thermo #K1622; K^+^ test Kitwere from Genmed; LDH colorimetric determination Kit were from Pulilai Gene technology Company; BCA protein assay Kit were from Jiangsu KeyGEN biology technology corp limited company; Protein pre-dyeing marker were from Thermo; The pvdf film were from Millipore; Tween-20 were from Amresco.

### Cells culture and identify

The diarrhea-predominant irritable bowel syndrome (IBS-D) rats models were induced by rectal distention pressure combining with extremities constraint. Rectal distention pressure (for 2 weeks): The balloon volume was 1.6 ml (hydrostatic pressure in Bowman’s space > 60 mm Hg) and lasted for 60 s, and for twice one day. Extremities constraint (for the next 5 days): Rats were put into the brake cage, tied front shoulders, front arms and chest, limited their scratch to the head and face, for 2 hours. The rats’ visceral sensitivity was evaluated by abdominal withdraw reaction on the third day after the modeling [26]. Rats(normal rats and IBS rats) colonic epithelial cells were cultured in this study. The rats colon tissues were taken and cleaned. The micro-villus on the colonic mucosal surface were treated with curettage, and the tissues were cut into the pieces of 1mm^3^ and put into the centrifugation tube(50ml). for 5 times after the PBS added. Standing for 30minutes and sucked out the PBS, then digested by the mixture of digestive fluid with 0.1% IV type collagenase and 0.1% I Type collagenase for 30 minutes(37°C). Beat up for 5 minutes and standing for 1 minutes. After that, the culture fluid was added. Repeated the standing and centrifugalization for 2-3 times. After 5-6 times centrifugation(1000rad/min), the culture fluid added and the cells were grown in the incubator(5%CO_2_, 37 °C). The colonic epithelial cells were identified. The cells were grown and staining. Fixed by Paraformaldehyde for 10 minutes Then reacted respectively with primary antibodies(Cytokeratin 19) and secondary antibodies(Anti-Mouse IgG). Then reacted with hoechst. After the seal, observed by microscope, red fluorescence was positive.

### Group of experiments

The cells were divided into four groups. Group A:normal rats primary colonic epithelial cells. Group B: IBS-D rats primary colonic epithelial cells. Group C: IBS-D rats primary colonic epithelial cells +miR-29a NC(negative control group relative to group D, which hardly influence the expression of miR-29a). Group D: IBS-D rats primary colonic epithelial cells +miR-29a antagomir(antagomir were added to increase the expression of miR-29a).

### Evaluation of MiRNA-29a by qRT–PCR

Total RNA was extracted. Reversed transcriptase cDNA was based on the specification. Real-time PCR amplification system of MiRNA: miRNAcDNA 2 μL; 2×SYBR Green Mix 10μL; Forward Primer(10μM) 0.5 μL; Reverse Primer(10μM) 0.5 μL; DEPC water, add up to 20 μL. Amplification for 40 circles (95 °C,20 s,62 °C,30 s,72 °C,30 s). The quantitation of PCR products relatively was conducted by qRT-PCR detected the colonic epithelial cells MiR-29a expression changes.

### Intestinal membrane permeability testing

Normally, LDH and K^+^ can’t go through the intestinal membrane when it is complete. The destroy of membrane integrity will result in the increase of LDH and K^+^ leakage rates. We tested the LDH concentration out of the cells and K^+^ concentration in the cells to evaluate the intestinal membrane permeability. The colonic epithelial cells were cleaned and cracked. After centrifugalization, Reagent D, Reagent E and Reagent F were added respectively. The sample were put into the spectrophotometer, the concentration of K^+^ was determined by light absorption readings (OD).

After the colonic epithelial cells cracked, zymolyte and Oxidized coenzyme were mixed at 5:1, then added into the microplate. 2, 4-Dinitrophenylhydrazine was added into the sample. Ended the reaction, then the concentration of LDH was determined at wavelength of 440 nm.

### Evaluation of AQPs by western blot

Total proteins were extracted from rats’ colonic epithelial cells, which were lysed with radio-immunoprecipitation assay buffer to which phosphatase inhibitor was added immediately after addition of the lysis buffer. The protein levels were evaluated with a BCA assay kit. Proteins were separated by SDS-polyacrylamide gel, and transferred to a NC membrane, then blocked in 5% skim milk. Target proteins were detected by corresponding primary antibodies (iNOS, TNF-α, IL-10, LC3B and GAPDH), and subsequently by horseradish peroxidase-conjugated secondary antibodies. Protien bands were analyzed and quantified the protein bands with gel image analyzer.

### Statistical analysis

Analysis were carried out by running the SPSS 18.0 statistical software. All data were normally distributed. Comparisons between groups were performed by Student's unpaired t test. Multiple comparisons within groups were performed by repeated measures one way analysis of variance, followed by Student's t test. Statistical significance was accepted at p<0.05.

## Abbreviations

IBS-D: diarrhea-predominant irritable bowel syndrome
miR-29a: microRNA-29a
AQPs: aquaporins
miRNAs: MicroRNAs
LDH: lactate dehydrogenase

## References

[1] Basseri RJ, Weitsman S, Barlow GM, Pimentel M (2011). Antibiotics for the treatment of irritable bowel syndrome. Gastroenterol Hepatol (NY).

[2] Verne GN, Himes NC, Robinson ME, Gopinath KS, Briggs RW, Crosson B, Price DD (2003). Central representation of visceral and cutaneous hypersensitivity in the irritable bowel syndrome. Pain.

[3] Verne GN, Robinson ME, Price DD (2001). Hypersensitivity to visceral and cutaneous pain in the irritable bowel syndrome. Pain.

[4] Lee YJ, Park KS (2014). Irritable bowel syndrome: emerging paradigm in pathophysiology. World J Gastroenterol.

[5] Bjarnason I, MacPherson A, Hollander D (1995). Intestinal permeability: an overview. Gastroenterology.

[6] Dunlop SP, Hebden J, Campbell E, Naesdal J, Olbe L, Perkins AC, Spiller RC (2006). Abnormal intestinal permeability in subgroups of diarrhea-predominant irritable bowel syndromes. Am J Gastroenterol.

[7] Spiller RC, Jenkins D, Thornley JP, Hebden JM, Wright T, Skinner M, Neal KR (2000). Increased rectal mucosal enteroendocrine cells, T lymphocytes, and increased gut permeability following acute Campylobacter enteritis and in postdysenteric irritable bowel syndrome. Gut.

[8] Spiller RC (2007). Role of infection in irritable bowel syndrome. J Gastroenterol.

[9] Zhou Q, Zhang B, Verne GN (2009). Intestinal membrane permeability and hyper-sensitivity in the irritable bowel syndrome. Pain.

[10] Martínez C, Vicario M, Ramos L, Lobo B, Mosquera JL, Alonso C, Sánchez A, Guilarte M, Antolín M, de Torres I, González-Castro AM, Pigrau M, Saperas E (2012). The jejunum of diarrhea-predominant irritable bowel syndrome shows molecular alterations in the tight junction signaling pathway that are associated with mucosal pathobiology and clinical manifestations. Am J Gastroenterol.

[11] Ishibashi K, Hara S, Kondo S (2009). Aquaporin water channels in mammals. Clin Exp Nephrol.

[12] Hara-Chikuma M, Verkman AS (2006). Physiological roles of glycerol transporting aquaporins: the aquaglyceroporins. Cell Mol Life Sci.

[13] Umberto Laforenza U (2012). Water channel proteins in the gastrointestinal tract. Mol Aspects Med.

[14] Zhao GX, Dong PP, Peng R, Li J, Zhang DY, Wang JY, Shen XZ, Dong L, Sun JY (2016). Expression, localization and possible functions of aquaporins 3 and 8 in rat digestive system. Biotech Histochem.

[15] Wang JP, Hou XH, Ma RJ (2006). The clinical features and colonic epithelium AQP8 expression in diarrhea-irritable bowel syndrome. [Article in Chinese] Zhonghua Nei Ke Za Zhi.

[16] Zhang W, Xu Y, Chen Z, Xu Z, Xu H (2011). Knockdown of aquaporin 3 is involved in intestinal barrier integrity impairment. FEBS Lett.

[17] Bartel DP (2004). MicroRNAs: genomics, biogenesis, mechanism, and function. Cell.

[18] O Connell RM, Rao DS, Baltimore D (2012). MicroRNA regulation of inflammatory responses. Annu Rev Immunol.

[19] Mansour MA, Sabbah NA, Mansour SA (2016). Ibrahim AM.MicroRNA-199b expression level and coliform count in irritable bowel syndrome. IUBMB Life.

[20] Liao XJ, Mao WM, Wang Q, Yang GG, Wu WJ, Shao SX (2016). MicroRNA-24 inhibits serotonin reuptake transporter expression and aggravates irritable bowel syndrome. Biochem Biophys Res Commun.

[21] Fourie NH, Peace RM, Abey SK, Sherwin LB, Rahim-Williams B, Smyser PA, Wiley JW, Henderson WA (2014). Elevated circulating miR-150 and miR-342-3p in patients with irritable bowel syndrome. Exp Mol Pathol.

[22] Zhou Q, Yang L, Larson S, Basra S, Merwat S, Tan A, Croce C, Verne GN (2016). Decreased miR-199 augments visceral pain in patients with IBS through translational upregulation of TRPV1. Gut.

[23] Vicario M, Martínez C, Santos J (2010). Role of microRNA in IBS with increased gut permeability. Gut.

[24] Zhou Q, Costinean S, Croce CM, Brasier AR, Merwat S, Larson SA, Basra S, Verne GN (2015). MicroRNA 29 targets nuclear factor-κB-repressing factor and Claudin 1 to increase intestinal permeability. Gastroenterology.

[25] Zhou Q, Souba WW, Croce CM, Verne GN (2010). MicroRNA-29a regulates intestinal membrane permeability in patients with irritable bowel syndrome. Gut.

[26] AL-Chaer ED, Kawasaki M, Pasricha PJ (2000). A new medel of chronic visceral hypersensitivity in adult rats induced by colon irritation during postnatal development. Gastroenterology.

[27] Sánchez de Medina F, Romero-Calvo I, Mascaraque C, Martínez-Augustin O (2014). Intestinal inflammation and mucosal barrier function. Inflamm Bowel Dis.

[28] Suzuki T (2013). Regulation of intestinal epithelial permeability by tight junctions. Cell Mol Life Sci. CMLS.

[29] Camilleri M, Gorman H (2007). Intestinal permeability and irritable bowel syndrome. Neurogastroenterol Motil.

[30] Camilleri M, Lasch K, Zhou W (2012). Irritable bowel syndrome: methods, mechanisms, and pathophysiology. The confluence of increased permeability, inflammation, and pain in irritable bowel syndrome. Am J Physiol Gastrointest Liver Physiol.

[31] Ishibashi K, Kondo S, Hara S, Morishita Y (2011). The evolutionary aspects of aquaporin family. Am J Physiol Regul Integr Comp Physiol.

[32] Liu HH, Liu XD, Wang YJ, Guan HQ, Chai JY, Zhao JR, Wang DS (2012). Effects of acupoint area and non-acupoint area of eye-acupuncture on expressions of VIP and AQP 8 in colonic tissues in rats with D-IBS. [Article in Chinese] Zhongguo Zhen Jiu.

[33] Hu R, Zhang T, Tang F (2010). Effect of Weichang’an pill on intestinal digestion enzymes and the AQP4 concentration in proximal colon in IBS-D rats. [Article in Chinese] Zhongguo Zhong Yao Za Zhi.

[34] Garson R, Marcucci G, Croce CM (2010). Targeting microRNAs in cancer: rationale, strategies and challenges. Nat Rev Drug Discov.

