# Correction: MicroRNA-29a increased the intestinal membrane permeability of colonic epithelial cells in irritable bowel syndrome rats

**DOI:** 10.18632/oncotarget.24848

**Published:** 2018-03-20

**Authors:** Guanqun Chao, Yingying Wang, Shuo Zhang, Weilin Yang, Zheying Ni, Xuliang Zheng

**Affiliations:** ^1^ Department of Family Medicine, Sir Run Run Shaw Hospital, Zhejiang University, China; ^2^ Department of Gastroenterology, The First Affiliated Hospital, Zhejiang Chinese Medical University, China

**This article has been corrected:** The proper name of the institutions are as follows:

^**1**^ Department of Family Medicine, Sir Run Run Shaw Hospital, Zhejiang University, China

^**2**^ Department of Gastroenterology, The First Affiliated Hospital, Zhejiang Chinese Medical University, China

Original article: Oncotarget. 2017; 8:85828-85837. https://doi.org/10.18632/oncotarget.20687

